# Supplementary material for: Unequal Recovery in Colorectal Cancer Screening Following the COVID-19 Pandemic: A Comparative Microsimulation Analysis
Source: medRxiv. 2022 Dec 26:2022.12.23.22283887. Preprint. [Version 1] doi: 10.1101/2022.12.23.22283887 (PMC9810216; doi:10.1101/2022.12.23.22283887)
Supplement: 1 [file NIHPP2022.12.23.22283887v1-supplement-1.pdf]

medRxiv preprint doi: https://doi.org/10.1101/2022.12.23.22283887; this version posted December 26, 2022. The copyright holder for this preprint (which was not certified by peer review) is the author/funder, who has granted medRxiv a license to display the preprint in perpetuity.

It is made available under a CC-BY 4.0 International license .

Supplementary Appendix

This supplementary appendix presents details about the computing environment used in the study, supplementary figures, and tables.

Computing Environment

All experiments were run on the Argonne Leadership Computing Facility's Theta supercomputer, and on Bebop, an HPC cluster managed by the Laboratory Computing Resource Center at Argonne National Laboratory, using the EMEWS workflow framework. Theta is a Cray XC40 with 4,392 compute nodes, each with an Intel KNL 7230 (Xeon Phi), aggregating 11.7 petaflops in total. Each node has 64 compute cores with access to 16 GB of high-bandwidth in-package memory, 192 GB of DDR4 RAM, and 128 GB of SSD. Bebop has 1024 nodes comprised of 672 Intel Broadwell processors with 36 cores per node and 128 GB of RAM and 372 Intel Knights Landing processors with 64 cores per node and 96 GB of RAM.

medRxiv preprint doi: https://doi.org/10.1101/2022.12.23.22283887; this version posted December 26, 2022. The copyright holder for this preprint (which was not certified by peer review) is the author/funder, who has granted medRxiv a license to display the preprint in perpetuity.

It is made available under a CC-BY 4.0 International license .

Supplementary Figure 1. Estimated loss of life in minor disruption scenarios

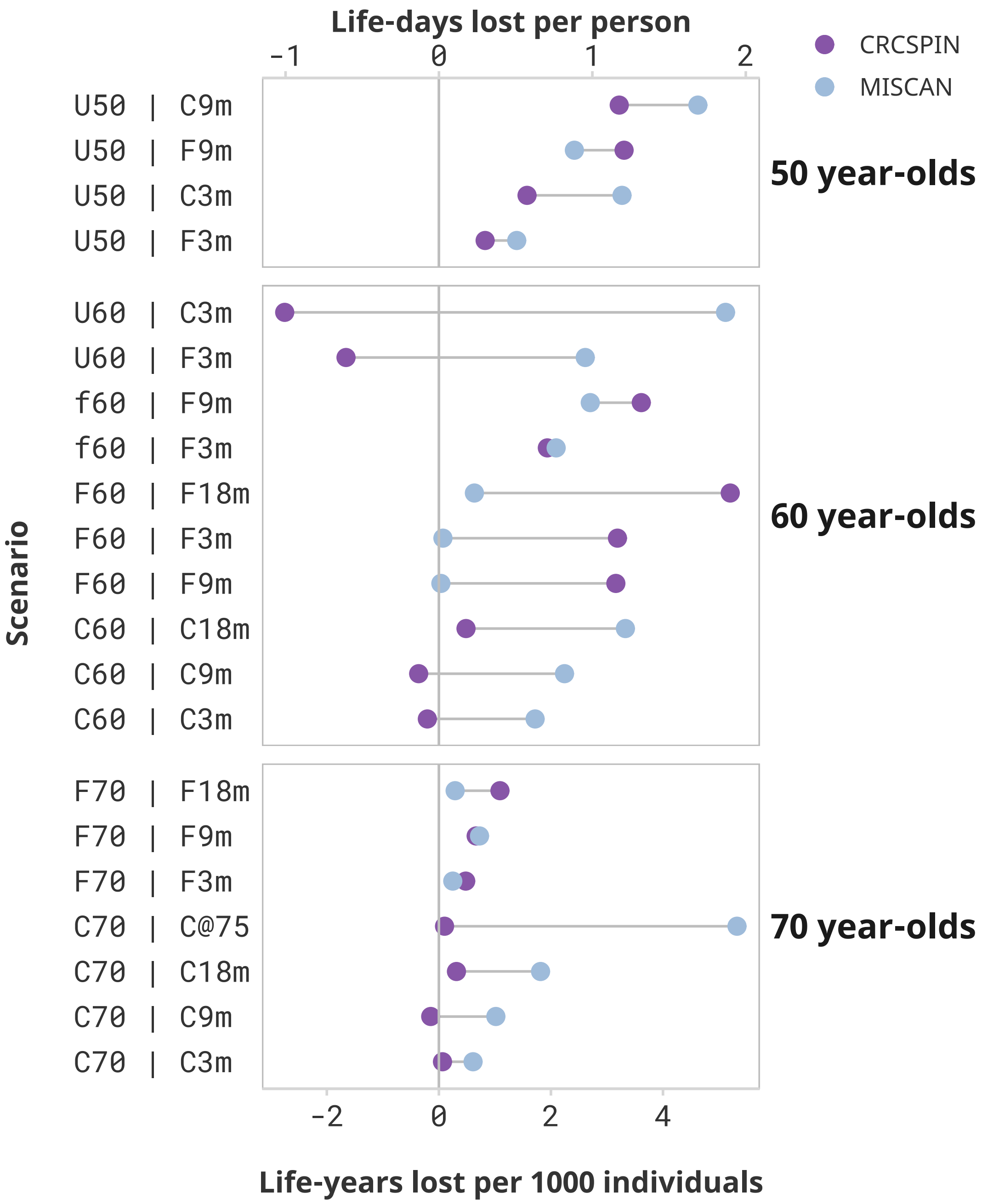

Notes: Each dot represents the estimated life-years lost per 1000 individuals or life-days lost from one model. Results are ordered from highest to lowest reduction in benefit induced by the pandemic

medRxiv preprint doi: https://doi.org/10.1101/2022.12.23.22283887; this version posted December 26, 2022. The copyright holder for this preprint (which was not certified by peer review) is the author/funder, who has granted medRxiv a license to display the preprint in perpetuity.

It is made available under a CC-BY 4.0 International license .

Supplementary Figure 2. Estimated loss of life under low sensitivity assumptions

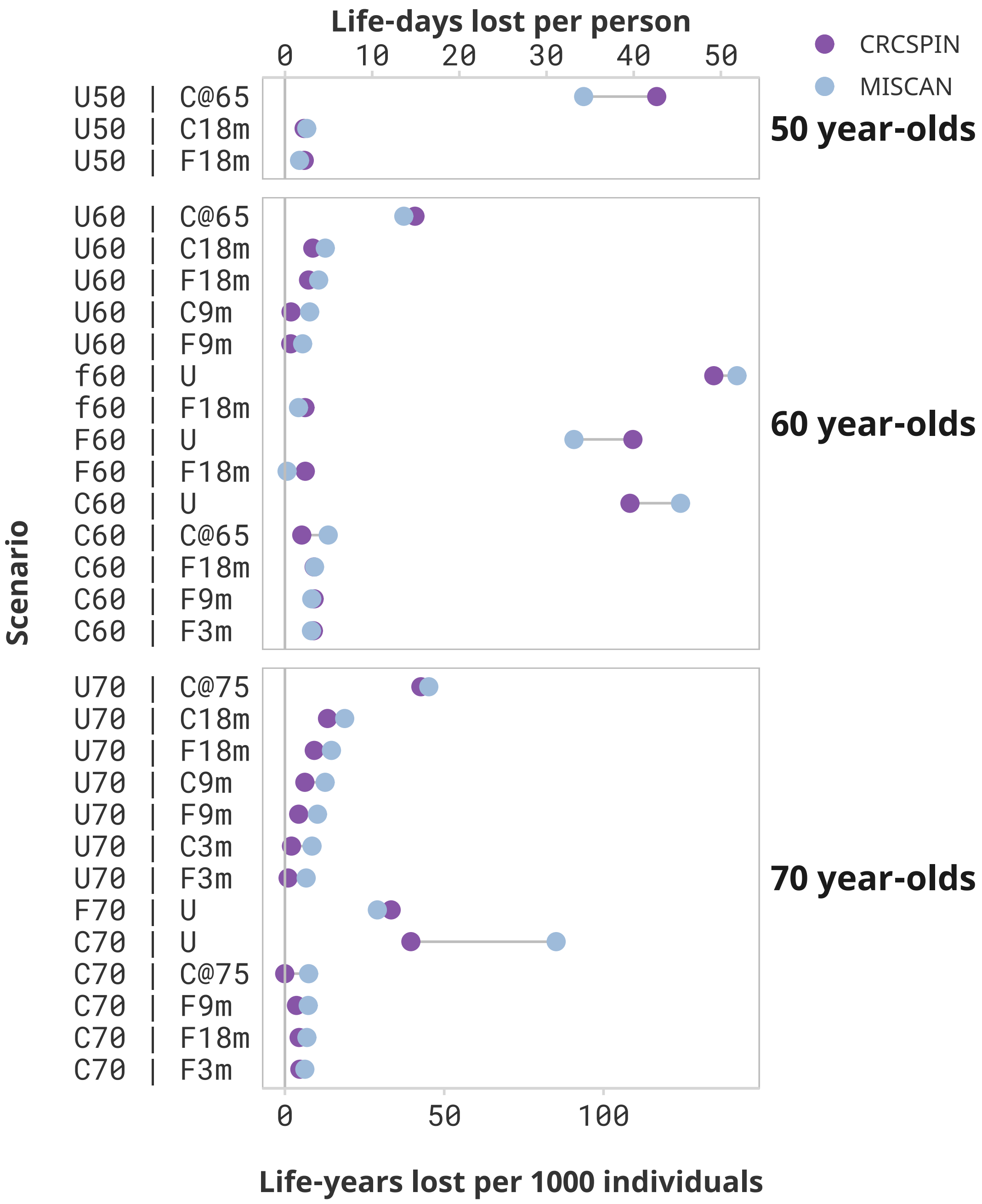

Notes: Each dot represents the estimated life-years lost per 1000 individuals or life-days lost from one model. Results are ordered from highest to lowest reduction in benefit induced by the pandemic.

medRxiv preprint doi: https://doi.org/10.1101/2022.12.23.22283887; this version posted December 26, 2022. The copyright holder for this preprint (which was not certified by peer review) is the author/funder, who has granted medRxiv a license to display the preprint in perpetuity.

It is made available under a CC-BY 4.0 International license .

Supplementary Figure 3. Estimated loss of life in minor disruption scenarios and low sensitivity assumptions

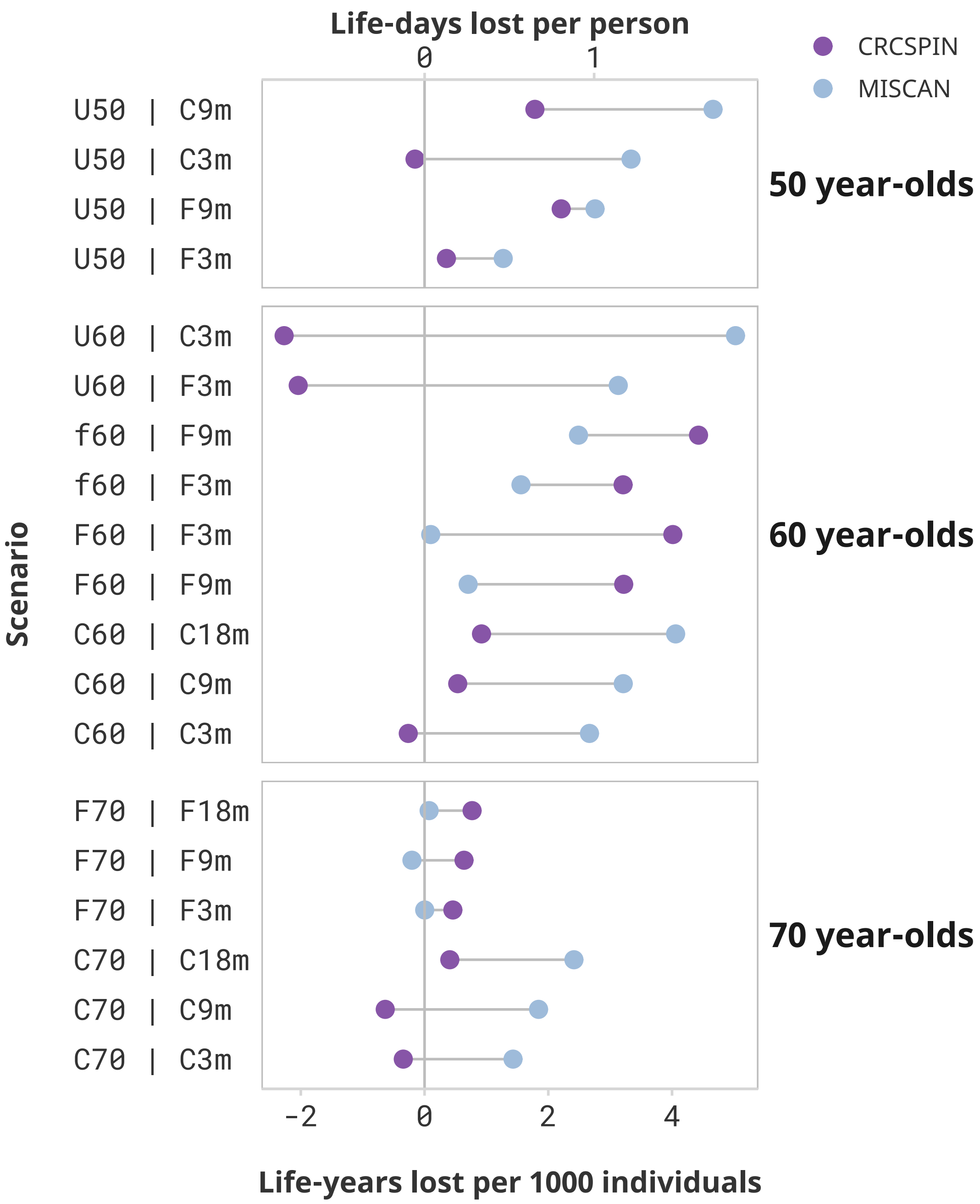

Notes: Each dot represents the estimated life-years lost per 1000 individuals or life-days lost from one model. Results are ordered from highest to lowest reduction in benefit induced by the pandemic.

medRxiv preprint doi: https://doi.org/10.1101/2022.12.23.22283887; this version posted December 26, 2022. The copyright holder for this preprint (which was not certified by peer review) is the author/funder, who has granted medRxiv a license to display the preprint in perpetuity.

It is made available under a CC-BY 4.0 International license .

Supplementary Figure 4. Life-years gained in high- vs. low-sensitivity scenarios

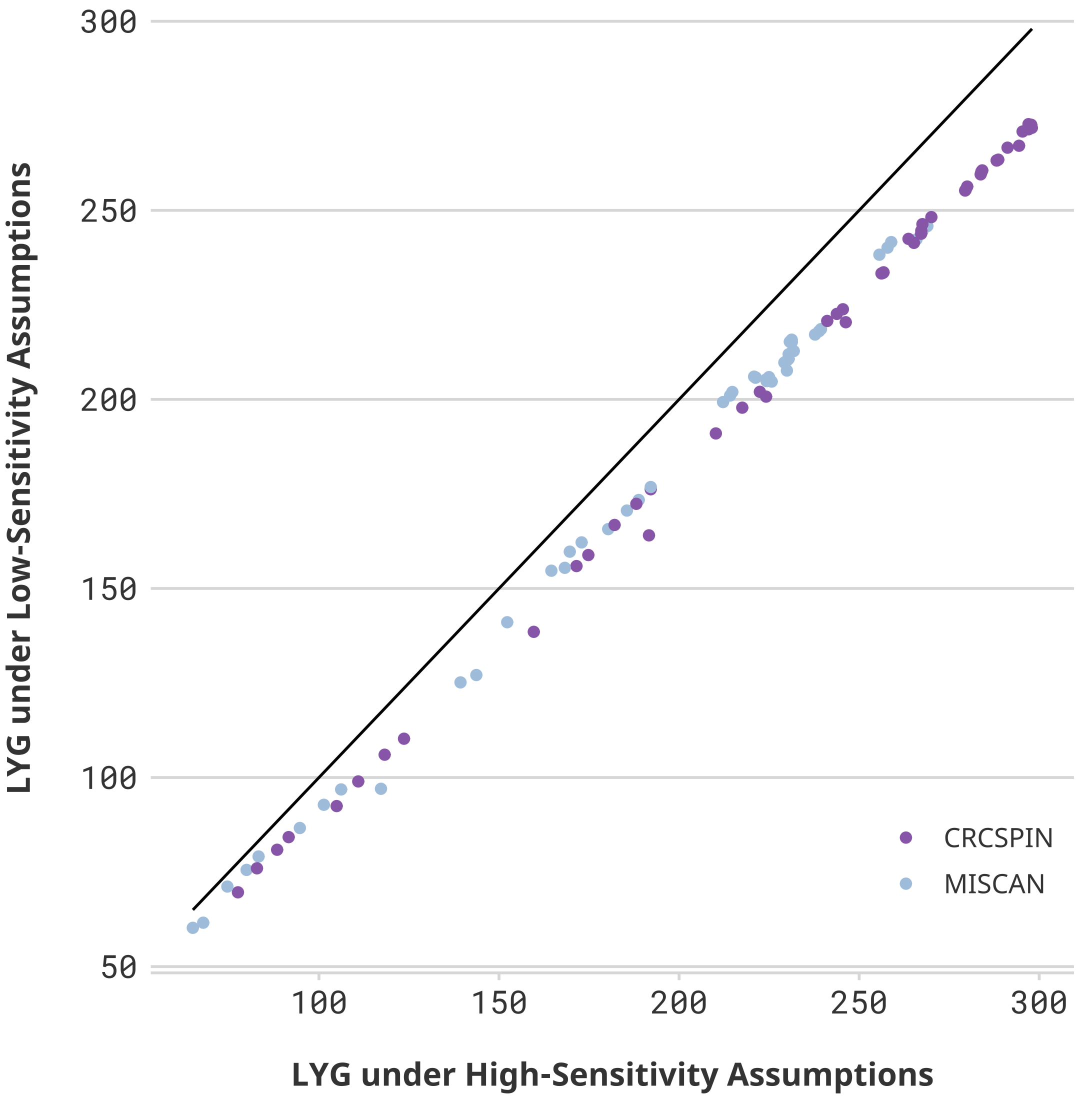

Notes: Each dot represents one scenario considered in this study. The horizontal axis displays the number of Life-years gained (LYG) estimated in that scenario under a high colonoscopy sensitivity scenario. The vertical axis shows the results for the same cohort under a low colonoscopy sensitivity scenario. If Sensitivity did not affect the estimate, then all points would be on top of a 45-degree line. Different colors represent CRCSPIN and MISCAN models.

medRxiv preprint doi: https://doi.org/10.1101/2022.12.23.22283887; this version posted December 26, 2022. The copyright holder for this preprint (which was not certified by peer review) is the author/funder, who has granted medRxiv a license to display the preprint in perpetuity.

It is made available under a CC-BY 4.0 International license .

Supplementary Figure 5. Life-years lost in high- vs. low-sensitivity scenarios

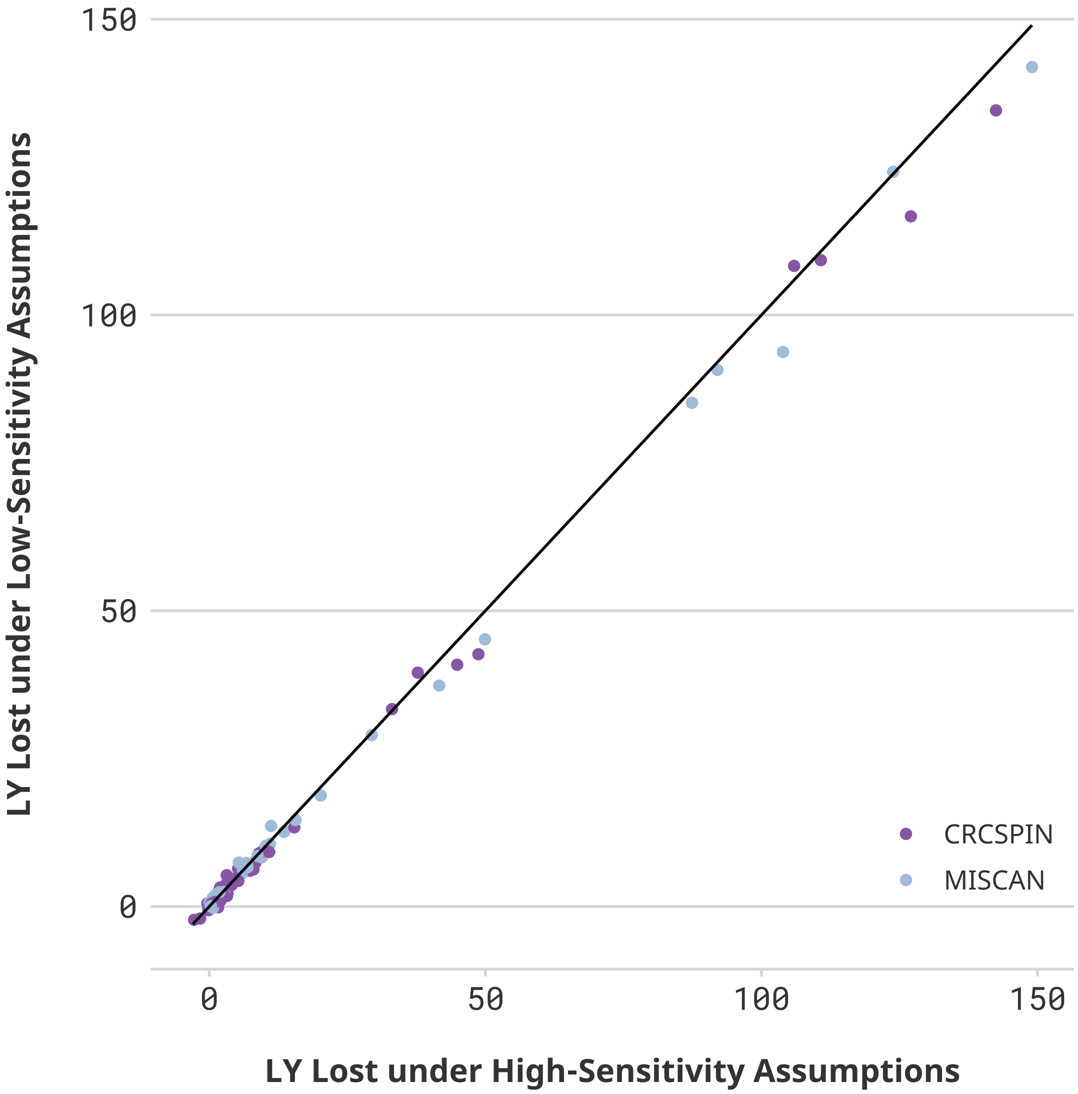

Notes: Each dot represents one scenario considered in this study. The horizontal axis displays the number of Life-years lost due to disruptions (LYL) estimated in that scenario under a high colonoscopy sensitivity scenario. The vertical axis shows the results for the same cohort under a low colonoscopy sensitivity scenario. If Sensitivity did not affect the estimate, then all points would be on top of a 45-degree line. Different colors represent CRCSPIN and MISCAN models.

medRxiv preprint doi: https://doi.org/10.1101/2022.12.23.22283887; this version posted December 26, 2022. The copyright holder for this preprint (which was not certified by peer review) is the author/funder, who has granted medRxiv a license to display the preprint in perpetuity.

It is made available under a CC-BY 4.0 International license .

Test Characteristics

This appendix specifies sensitivity and specificity assumptions underlying colonoscopy and FIT exams evaluated in this study. Supplementary Table 2 specifies per-lesion sensitivity and specificity assumptions for the two scenarios evaluated in this paper.

Supplementary Table 1. Per lesion test sensitivity and specificity

Test	Sensitivity*				Specificity **
	Adenoma 1-5mm	Adenoma 6-9mm	Adenoma ≥10mm	Preclinical cancer	
Colonoscopy, high sensitivity†	0.75	0.85	0.95	0.95	0.86
Colonoscopy, low sensitivity‡	0.55	0.70	0.90	0.95	0.86
FIT§					
MISCAN	0.00	0.114	0.159	0.62565/0.886	0.97
CRC-SPIN	0.05	0.15	0.22	*0.74	0.97

Notes: This table presents the assumed test characteristics. We simulated two colonoscopy sensitivity scenarios seeking to represent a range of colonoscopy sensitivity of gastroenterologists in the US.
*Sensitivity is for lesions within reach of the scope. We assume the same test characteristics for follow-up and surveillance colonoscopy as for screening colonoscopy.
**For FIT tests, the lack of specificity reflects detection of bleeding from other causes. We assume other-cause bleeding is independent of adenoma status. For colonoscopy, the lack of specificity reflects detection of non-adenomatous lesions, but specificity is handled in post-processing in cost-effectiveness analyses. Since this study does not consider burden outcomes, specificity is not considered in this paper. Specificity values were obtained from Lin et al.[26]
† Baseline scenarios used in Zauber et al.[27]
‡ In line with low-sensitivity scenarios compatible with Rutter et al.[28]
§ CRC-SPIN uses per-person test sensitivity for stool-based tests that are based on the size of the most advanced lesion. To account for the likelihood that a person with multiple adenomas is more likely than a person with only one to have a positive stool test, MISCAN uses lesion-based sensitivities instead of person-based sensitivities. Lesion-based sensitivities were derived by calibrating the person-based sensitivities to the number of people having one or more small/medium/large adenomas or cancers detected by stool-based testing with diagnostic colonoscopy, divided by those having one or more small/medium/large adenomas or cancers detected by colonoscopy screening.

medRxiv preprint doi: https://doi.org/10.1101/2022.12.23.22283887; this version posted December 26, 2022. The copyright holder for this preprint (which was not certified by peer review) is the author/funder, who has granted medRxiv a license to display the preprint in perpetuity.

It is made available under a CC-BY 4.0 International license .

CRC Surveillance

We assume that individuals with an adenoma detected undergo colonoscopic surveillance according to the Multi-Society Task Force (MSTF) guidelines. These guidelines provide intervals for surveillance based on baseline findings and findings at the first surveillance colonoscopy. We assume the intervals provided can be more generally expressed as the intervals based on the most recent colonoscopy ("first most recent colonoscopy") and the colonoscopy prior to that ("second most-recent colonoscopy"). In situations where the MSTF provided a range rather than a single interval, we assumed that the shortest interval would be used in routine practice. The resulting intervals are shown in **Supplementary Table 3**.

We assume that persons in whom adenoma(s) have been detected remain on surveillance until age 85, provided that no adenomas are detected at the last surveillance colonoscopy. If adenomas are detected, then surveillance continues according to the clinical findings at the last colonoscopy until the person has a colonoscopy with no adenomas detected. For example, if a person has a surveillance colonoscopy at age 83 and no adenomas are detected at this exam or the exam before this one, they would be recommended to have their next surveillance at age 93. Age 93 is after the surveillance stopping age of 85 and the exam prior to age 85 was negative, so they will not have any more surveillance colonoscopies after age 83. However, if the exam at age 83 instead detected 1-2 small adenomas, they would come back for their surveillance colonoscopy at age 90, because adenomas were detected at the exam at age 83. As noted in the section on adherence above, in the primary analyses, we assume persons with adenoma findings are perfectly adherent with the surveillance colonoscopy schedules shown in **Supplementary Table 3**.

medRxiv preprint doi: https://doi.org/10.1101/2022.12.23.22283887; this version posted December 26, 2022. The copyright holder for this preprint (which was not certified by peer review) is the author/funder, who has granted medRxiv a license to display the preprint in perpetuity.
It is made available under a CC-BY 4.0 International license .

Supplementary Table 2. CRC Surveillance Intervals

Finding at second-most recent colonoscopy*†	Finding at first-most recent colonoscopy*†	Interval‡ to next colonoscopy, y
No prior colonoscopy	Normal colonoscopy	See note below§
	1-2 adenomas <10 mm	7
	3-4 adenomas <10 mm	3
	10 adenomas <10 mm or any adenoma ≥10 mm	3
	> 10 adenomas	1
Normal colonoscopy	Normal colonoscopy	10
	1-2 adenomas <10 mm	7
	3-4 adenomas <10 mm	3
	5-10 adenomas <10 mm or any adenoma ≥10 mm	3
	> 10 adenomas	1
1-2 adenomas <10 mm	Normal colonoscopy	10
	1-2 adenomas <10 mm	7
	3-4 adenomas <10 mm	3
	5-10 adenomas <10 mm or any adenoma ≥10 mm	3
	> 10 adenomas	1
3-4 adenomas <10 mm	Normal colonoscopy	10
	1-2 adenomas <10 mm	7
	3-4 adenomas <10 mm	3
	5-10 adenomas <10 mm or any adenoma ≥10 mm	3
	> 10 adenomas	1
5-10 adenomas <10 mm or any adenoma ≥10 mm	Normal colonoscopy	5
	1-2 adenomas <10 mm	5
	3-4 adenomas <10 mm	3
	5-10 adenomas <10 mm or any adenoma ≥10 mm	3
	> 10 adenomas	1
> 10 adenomas of any size	Normal colonoscopy	5
	1-2 adenomas <10 mm	5
	3-4 adenomas <10 mm	3
	5-10 adenomas <10 mm or any adenoma ≥10 mm	3
	>10 adenomas	1

* A normal colonoscopy is one in which no adenomas, SSPs (not currently simulated), or CRC is detected.

† This table omits the case where CRC is detected at a screening, diagnostic, or surveillance colonoscopy because the CISNET CRC models do not simulate detailed events following CRC diagnosis.

‡ The Multi-Society Task Force provides a range for some intervals (e.g., the interval for 3-4 adenomas <10 mm is 3-5 years). In such cases, we selected the shortest intervals provided.

§ A person whose first screening or diagnostic colonoscopy is normal does not enter surveillance but instead resumes screening with the original modality 10 years after the normal colonoscopy. The exception to the 10-year waiting period is when the first colonoscopy is a screening colonoscopy with an *x*-year interval, where *x* >10. In that case, the next colonoscopy is in *x* years.

Supplementary Table 3. Lifetime estimates of CRC outcomes

Colonoscopy Sensitivity	Scenario	Model	Life-Years (LY)	LY No Screening	LY No Disruptions	LYG No Disruptions	LYG Screening	LY Lost Disruptions	% LYG Loss
High	U50 | C3m	CRCSPIN	31892	31595	31893	299	297	2	1
		MISCAN	31424	31156	31428	272	269	3	1
	U50 | C9m	CRCSPIN	31890	31595	31893	299	295	3	1
		MISCAN	31423	31156	31428	272	268	5	2
	U50 | C18m	CRCSPIN	31886	31595	31893	299	291	7	2
		MISCAN	31421	31156	31428	272	266	6	2
	U50 | C@65	CRCSPIN	31766	31595	31893	299	172	127	43
		MISCAN	31324	31156	31428	272	168	104	38
	U50 | F3m	CRCSPIN	31865	31595	31866	271	270	1	0
		MISCAN	31414	31156	31416	260	259	1	1
	U50 | F9m	CRCSPIN	31862	31595	31866	271	268	3	1
		MISCAN	31413	31156	31416	260	258	2	1
	U50 | F18m	CRCSPIN	31858	31595	31866	271	264	7	3
		MISCAN	31411	31156	31416	260	256	5	2
	U60 | C3m	CRCSPIN	23440	23218	23438	220	222	-3	-1
		MISCAN	23139	22950	23144	194	189	5	3
	U60 | C9m	CRCSPIN	23435	23218	23438	220	218	2	1
		MISCAN	23136	22950	23144	194	186	8	4
	U60 | C18m	CRCSPIN	23428	23218	23438	220	210	9	4
		MISCAN	23131	22950	23144	194	180	14	7
	U60 | C@65	CRCSPIN	23393	23218	23438	220	175	45	20
		MISCAN	23103	22950	23144	194	152	42	21
	U60 | F3m	CRCSPIN	23410	23218	23408	190	192	-2	-1
		MISCAN	23123	22950	23126	176	173	3	1
	U60 | F9m	CRCSPIN	23406	23218	23408	190	188	2	1

Colonoscopy Sensitivity	Scenario	Model	Life-Years (LY)	LY No Screening	LY No Disruptions	LYG No Disruptions	LYG Screening	LY Lost Disruptions	% LYG Loss
	U60 | F18m	MISCAN	23120	22950	23126	176	170	6	3
		CRCSPIN	23400	23218	23408	190	182	8	4
	C60 | C3m	MISCAN	23115	22950	23126	176	165	11	6
		CRCSPIN	23483	23185	23482	298	298	0	0
	C60 | C9m	MISCAN	23163	22924	23165	241	239	2	1
		CRCSPIN	23483	23185	23482	298	298	0	0
	C60 | C18m	MISCAN	23163	22924	23165	241	239	2	1
		CRCSPIN	23482	23185	23482	298	297	0	0
	C60 | F3m	MISCAN	23161	22924	23165	241	238	3	1
		CRCSPIN	23474	23185	23483	298	289	9	3
	C60 | F9m	MISCAN	23155	22924	23165	241	232	10	4
		CRCSPIN	23473	23185	23483	298	288	9	3
	C60 | F18m	MISCAN	23155	22924	23165	241	232	9	4
		CRCSPIN	23473	23185	23482	298	288	9	3
	C60 | C@65	MISCAN	23154	22923	23165	241	230	11	4
		CRCSPIN	23479	23185	23483	298	294	3	1
	C60 | U	MISCAN	23154	22924	23165	241	230	11	5
		CRCSPIN	23377	23185	23483	298	192	106	36
	F60 | F3m	MISCAN	23041	22924	23165	241	117	124	51
		CRCSPIN	23457	23190	23460	270	267	3	1
	F60 | F9m	MISCAN	23157	22925	23157	231	231	0	0
		CRCSPIN	23456	23189	23460	270	267	3	1
	F60 | F18m	MISCAN	23156	22925	23156	231	231	0	0
		CRCSPIN	23454	23189	23460	270	265	5	2
	F60 | U	MISCAN	23156	22925	23157	231	231	1	0
		CRCSPIN	23349	23189	23460	270	160	111	41

Colonoscopy Sensitivity	Scenario	Model	Life-Years (LY)	LY No Screening	LY No Disruptions	LYG No Disruptions	LYG Screening	LY Lost Disruptions	% LYG Loss
	f60 | F3m	MISCAN	23064	22925	23156	231	139	92	40
		CRCSPIN	23440	23194	23442	247	245	2	1
	f60 | F9m	MISCAN	23145	22930	23147	217	215	2	1
		CRCSPIN	23438	23194	23442	247	244	4	1
	f60 | F18m	MISCAN	23145	22931	23147	217	214	3	1
		CRCSPIN	23436	23194	23442	247	241	6	3
	f60 | U	MISCAN	23143	22931	23147	217	212	5	2
		CRCSPIN	23299	23194	23442	247	105	142	58
		MISCAN	22998	22930	23147	217	68	149	69
	U70 | C3m	CRCSPIN	15849	15725	15851	126	124	3	2
	U70 | C9m	MISCAN	15550	15444	15559	115	106	9	8
		CRCSPIN	15843	15725	15851	126	118	8	6
	U70 | C18m	MISCAN	15546	15444	15559	115	101	14	12
		CRCSPIN	15836	15725	15851	126	111	15	12
	U70 | C@75	MISCAN	15539	15444	15559	115	95	20	18
		CRCSPIN	15803	15725	15851	126	78	49	39
	U70 | F3m	MISCAN	15509	15444	15559	115	65	50	43
		CRCSPIN	15817	15725	15819	94	92	2	2
	U70 | F9m	MISCAN	15527	15444	15534	90	83	7	8
		CRCSPIN	15814	15725	15819	94	88	5	6
	U70 | F18m	MISCAN	15524	15444	15534	90	80	10	11
		CRCSPIN	15808	15725	15819	94	83	11	12
	C70 | C3m	MISCAN	15519	15444	15534	90	75	16	17
		CRCSPIN	15905	15621	15905	284	284	0	0
	C70 | C9m	MISCAN	15596	15366	15597	231	230	1	0
		CRCSPIN	15905	15621	15905	284	284	0	0

Colonoscopy Sensitivity	Scenario	Model	Life-Years (LY)	LY No Screening	LY No Disruptions	LYG No Disruptions	LYG Screening	LY Lost Disruptions	% LYG Loss
	C70 | C18m	MISCAN	15596	15366	15597	231	230	1	0
		CRCSPIN	15905	15621	15905	284	284	0	0
	C70 | C@75	MISCAN	15595	15366	15597	231	229	2	1
		CRCSPIN	15905	15621	15905	284	284	0	0
	C70 | F3m	MISCAN	15592	15366	15597	231	226	5	2
		CRCSPIN	15900	15621	15905	284	279	5	2
	C70 | F9m	MISCAN	15591	15366	15597	231	225	6	3
		CRCSPIN	15901	15621	15905	284	280	4	1
	C70 | F18m	MISCAN	15590	15366	15597	231	224	7	3
		CRCSPIN	15901	15621	15905	284	280	4	2
	C70 | U	MISCAN	15590	15366	15597	231	224	7	3
		CRCSPIN	15867	15621	15905	284	246	38	13
	F70 | F3m	MISCAN	15515	15372	15603	231	144	87	38
		CRCSPIN	15888	15631	15888	257	257	0	0
	F70 | F9m	MISCAN	15588	15367	15588	222	221	0	0
		CRCSPIN	15888	15631	15889	257	257	1	0
	F70 | F18m	MISCAN	15588	15367	15589	222	221	1	0
		CRCSPIN	15888	15632	15889	257	256	1	0
	F70 | U	MISCAN	15588	15367	15588	222	221	0	0
		CRCSPIN	15855	15631	15888	257	224	33	13
		MISCAN	15559	15367	15588	222	192	29	13
Low	U50 | C3m	CRCSPIN	31867	31595	31867	273	273	0	0
	U50 | C9m	MISCAN	31401	31156	31405	249	246	3	1
		CRCSPIN	31866	31595	31867	273	271	2	1
	U50 | C18m	MISCAN	31400	31156	31405	249	245	5	2
		CRCSPIN	31861	31595	31867	273	267	6	2

Colonoscopy Sensitivity	Scenario	Model	Life-Years (LY)	LY No Screening	LY No Disruptions	LYG No Disruptions	LYG Screening	LY Lost Disruptions	% LYG Loss
	U50 | C@65	MISCAN	31398	31156	31405	249	242	7	3
		CRCSPIN	31751	31595	31867	273	156	117	43
	U50 | F3m	MISCAN	31311	31156	31405	249	155	94	38
		CRCSPIN	31843	31595	31843	249	248	0	0
	U50 | F9m	MISCAN	31397	31156	31398	243	242	1	1
		CRCSPIN	31841	31595	31843	249	246	2	1
	U50 | F18m	MISCAN	31396	31156	31398	243	240	3	1
		CRCSPIN	31837	31595	31843	249	242	6	2
	U60 | C3m	MISCAN	31394	31156	31398	243	238	5	2
		CRCSPIN	23420	23218	23418	200	202	-2	-1
	U60 | C9m	MISCAN	23124	22950	23129	178	173	5	3
		CRCSPIN	23416	23218	23418	200	198	2	1
	U60 | C18m	MISCAN	23121	22950	23129	178	171	8	4
		CRCSPIN	23409	23218	23418	200	191	9	4
	U60 | C@65	MISCAN	23116	22950	23129	178	166	13	7
		CRCSPIN	23377	23218	23418	200	159	41	20
	U60 | F3m	MISCAN	23091	22950	23129	178	141	37	21
		CRCSPIN	23394	23218	23392	174	176	-2	-1
	U60 | F9m	MISCAN	23112	22950	23116	165	162	3	2
		CRCSPIN	23390	23218	23392	174	172	2	1
	U60 | F18m	MISCAN	23110	22950	23116	165	160	6	3
		CRCSPIN	23385	23218	23392	174	167	7	4
	C60 | C3m	MISCAN	23105	22950	23116	165	155	11	6
		CRCSPIN	23460	23188	23460	272	273	0	0
	C60 | C9m	MISCAN	23145	22926	23148	221	219	3	1
		CRCSPIN	23460	23188	23460	272	272	1	0
	C60 | C18m	MISCAN	23144	22926	23147	221	218	3	1
		CRCSPIN	23459	23188	23460	272	271	1	0

Colonoscopy Sensitivity	Scenario	Model	Life-Years (LY)	LY No Screening	LY No Disruptions	LYG No Disruptions	LYG Screening	LY Lost Disruptions	% LYG Loss
	C60 | F3m	MISCAN	23143	22926	23147	221	217	4	2
		CRCSPIN	23451	23188	23460	272	263	9	3
	C60 | F9m	MISCAN	23139	22926	23147	221	213	8	4
		CRCSPIN	23451	23188	23460	272	263	9	3
	C60 | F18m	MISCAN	23139	22926	23147	221	213	8	4
		CRCSPIN	23451	23188	23460	272	263	9	3
	C60 | C@65	MISCAN	23138	22926	23147	221	212	9	4
		CRCSPIN	23455	23188	23460	272	267	5	2
	C60 | U	MISCAN	23134	22926	23148	221	208	14	6
		CRCSPIN	23352	23187	23460	272	164	108	40
	F60 | F3m	MISCAN	23023	22926	23148	221	97	124	56
		CRCSPIN	23434	23191	23438	248	244	4	2
	F60 | F9m	MISCAN	23142	22926	23142	216	216	0	0
		CRCSPIN	23435	23191	23438	248	245	3	1
	F60 | F18m	MISCAN	23142	22926	23142	216	215	1	0
		CRCSPIN	23432	23191	23439	248	241	6	3
	F60 | U	MISCAN	23141	22926	23142	216	215	1	0
		CRCSPIN	23329	23191	23438	248	139	109	44
	f60 | F3m	MISCAN	23052	22926	23142	216	125	91	42
		CRCSPIN	23419	23195	23422	227	224	3	1
	f60 | F9m	MISCAN	23133	22931	23135	204	202	2	1
		CRCSPIN	23418	23195	23422	227	223	4	2
	f60 | F18m	MISCAN	23132	22931	23135	204	201	2	1
		CRCSPIN	23416	23195	23422	227	221	6	3
	f60 | U	MISCAN	23130	22931	23135	204	199	4	2
		CRCSPIN	23288	23195	23422	227	92	135	59
		MISCAN	22993	22931	23135	204	62	142	70
	U70 | C3m	CRCSPIN	15835	15725	15837	112	110	2	2

Colonoscopy Sensitivity	Scenario	Model	Life-Years (LY)	LY No Screening	LY No Disruptions	LYG No Disruptions	LYG Screening	LY Lost Disruptions	% LYG Loss
	U70 | C9m	MISCAN	15541	15444	15550	105	97	9	8
		CRCSPIN	15831	15725	15837	112	106	6	6
	U70 | C18m	MISCAN	15537	15444	15550	105	93	13	12
		CRCSPIN	15824	15725	15837	112	99	13	12
	U70 | C@75	MISCAN	15531	15444	15550	105	87	19	18
		CRCSPIN	15795	15725	15837	112	70	43	38
	U70 | F3m	MISCAN	15504	15444	15550	105	60	45	43
		CRCSPIN	15809	15725	15810	85	84	1	1
	U70 | F9m	MISCAN	15523	15444	15530	86	79	7	8
		CRCSPIN	15806	15725	15810	85	81	4	5
	U70 | F18m	MISCAN	15520	15444	15530	86	76	10	12
		CRCSPIN	15801	15725	15810	85	76	9	11
	C70 | C3m	MISCAN	15515	15444	15530	86	71	15	17
		CRCSPIN	15889	15629	15889	260	260	0	0
	C70 | C9m	MISCAN	15584	15373	15585	212	211	1	1
		CRCSPIN	15890	15629	15889	260	261	-1	0
	C70 | C18m	MISCAN	15583	15373	15585	212	210	2	1
		CRCSPIN	15889	15629	15889	260	260	0	0
	C70 | C@75	MISCAN	15583	15373	15585	212	210	2	1
		CRCSPIN	15889	15629	15889	260	260	0	0
	C70 | F3m	MISCAN	15578	15373	15585	212	205	7	4
		CRCSPIN	15885	15629	15889	260	255	5	2
	C70 | F9m	MISCAN	15579	15373	15585	212	206	6	3
		CRCSPIN	15886	15629	15889	260	256	4	1
	C70 | F18m	MISCAN	15578	15373	15586	212	205	7	3
		CRCSPIN	15885	15629	15889	260	255	4	2
	C70 | U	MISCAN	15578	15373	15585	212	205	7	3
		CRCSPIN	15850	15629	15889	260	220	40	15

Colonoscopy Sensitivity	Scenario	Model	Life-Years (LY)	LY No Screening	LY No Disruptions	LYG No Disruptions	LYG Screening	LY Lost Disruptions	% LYG Loss
	F70 | F3m	MISCAN	15505	15378	15591	212	127	85	40
		CRCSPIN	15871	15638	15872	234	234	0	0
	F70 | F9m	MISCAN	15577	15372	15577	206	206	0	0
		CRCSPIN	15872	15638	15872	234	233	1	0
	F70 | F18m	MISCAN	15578	15372	15578	206	206	0	0
		CRCSPIN	15871	15638	15872	234	233	1	0
	F70 | U	MISCAN	15577	15372	15577	206	206	0	0
		CRCSPIN	15839	15638	15872	234	201	33	14
		MISCAN	15548	15372	15577	206	177	29	14

Notes: Outcomes calculated over the lifetime of a cohort of 1000 average-risk, CRC-free individuals with age at pandemic defined in the scenario description. The scenario column describes colorectal cancer screening disruption scenarios, as presented in Table 1. The Life-years (LY) column presents life-years after 2020 under the specified scenarios. LY No Screening is a counterfactual scenario under which no CRC screening is performed. LY No Disruptions refers to a scenario of no pandemic-induced disruptions and no delays, and no switching of screening regimen. Life-years gained (LYG) under no disruptions refers to the number of life-years gained had no pandemic-induced disruptions happened. LYG Screening refers to the expected life-years gained under the specified scenario. LY Lost Pandemic refers to the number of life-years lost due to disruptions, and Perc LYG Loss refers to the percent reduction in LYG due to the pandemic relative to the no-disruption scenario. All values refer to cohort-level estimates – that is, the expected life-years of an average-risk person.

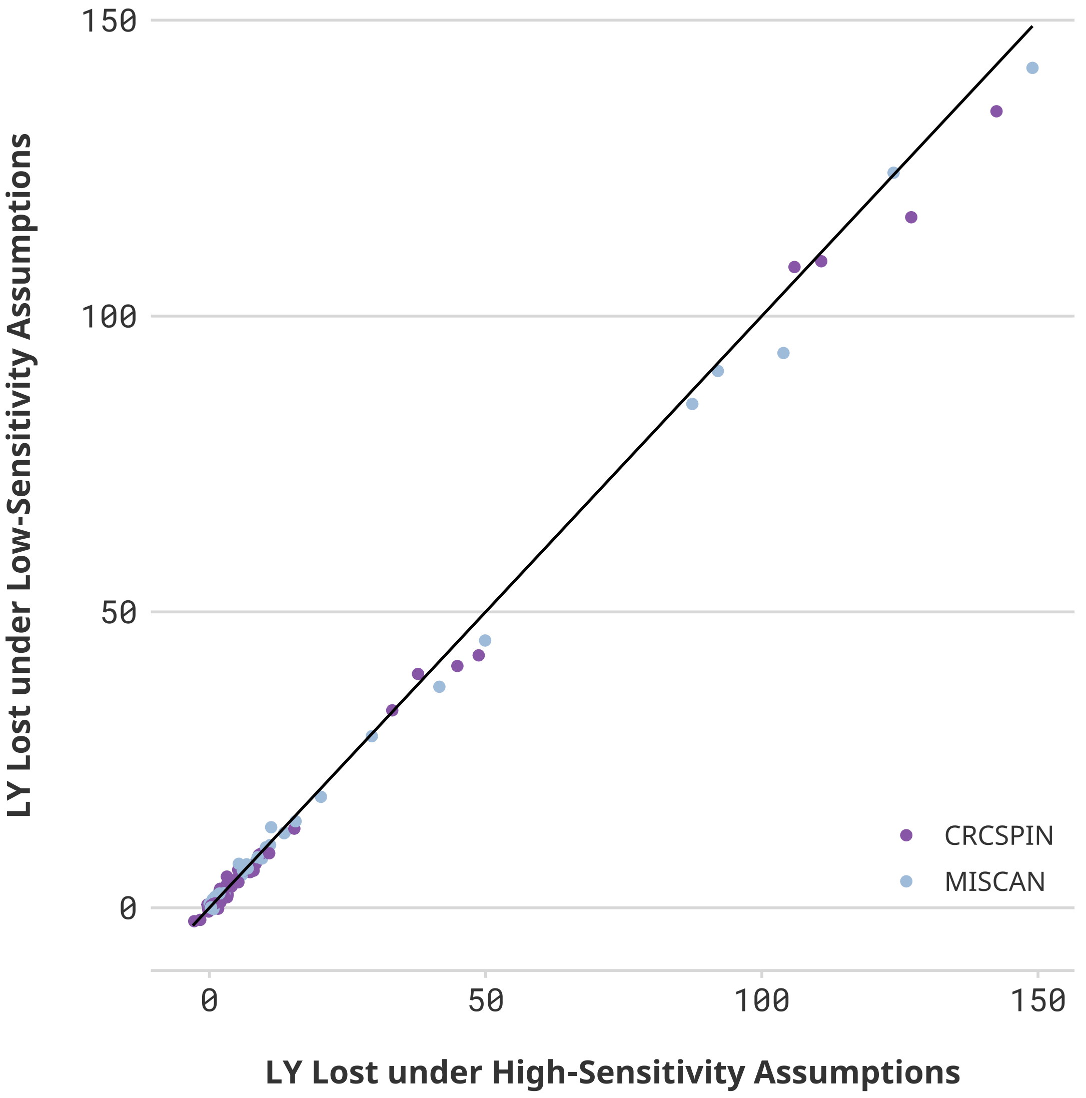

150
100
50
0
0
50
100
150
LY Lost under Low-Sensitivity Assumptions
LY Lost under High-Sensitivity Assumptions
CRCSPIN
MISCAN

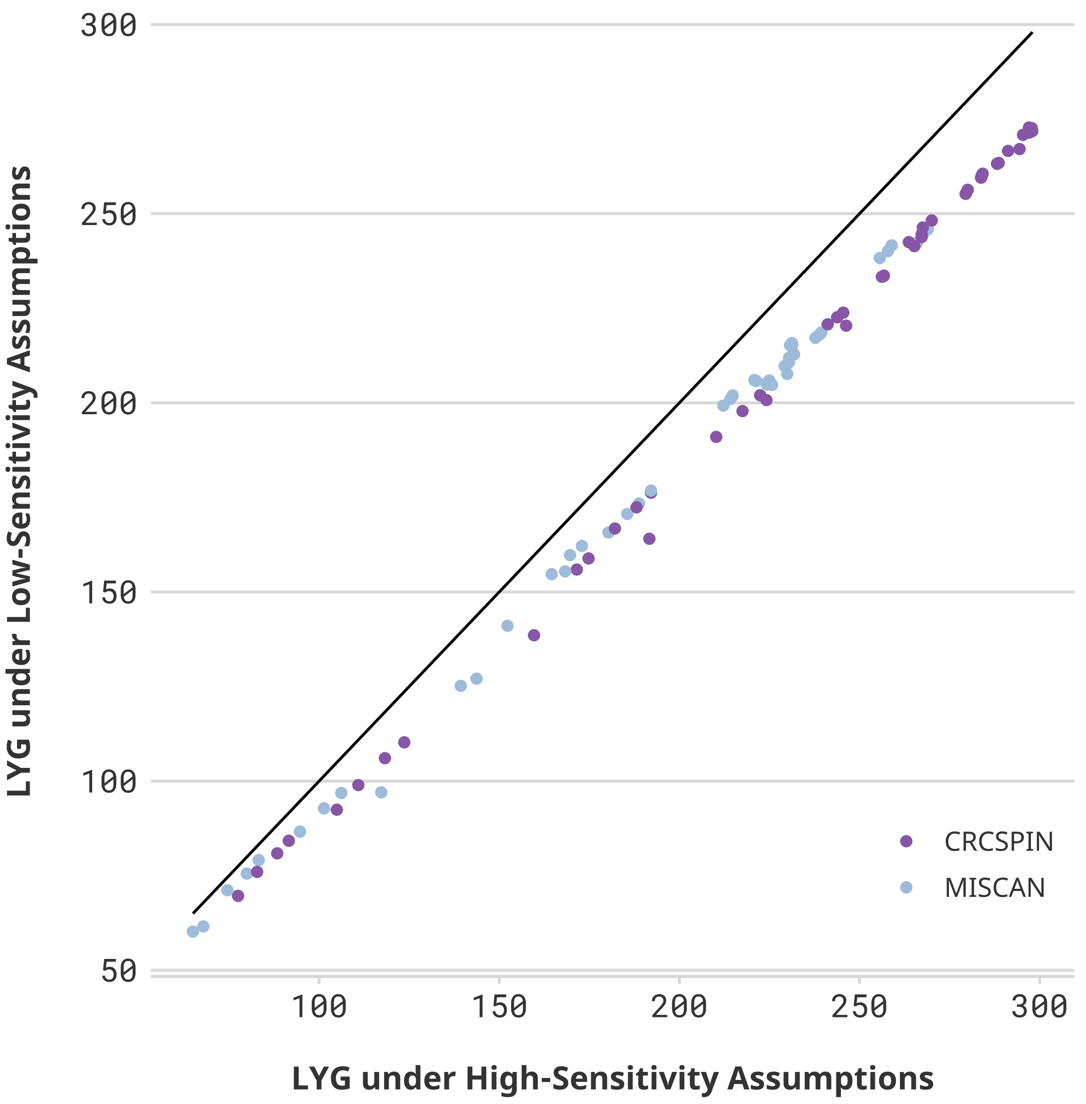

LYG under Low-Sensitivity Assumptions
LYG under High-Sensitivity Assumptions
300
250
200
150
100
50
100
150
200
250
300
CRCSPIN
MISCAN

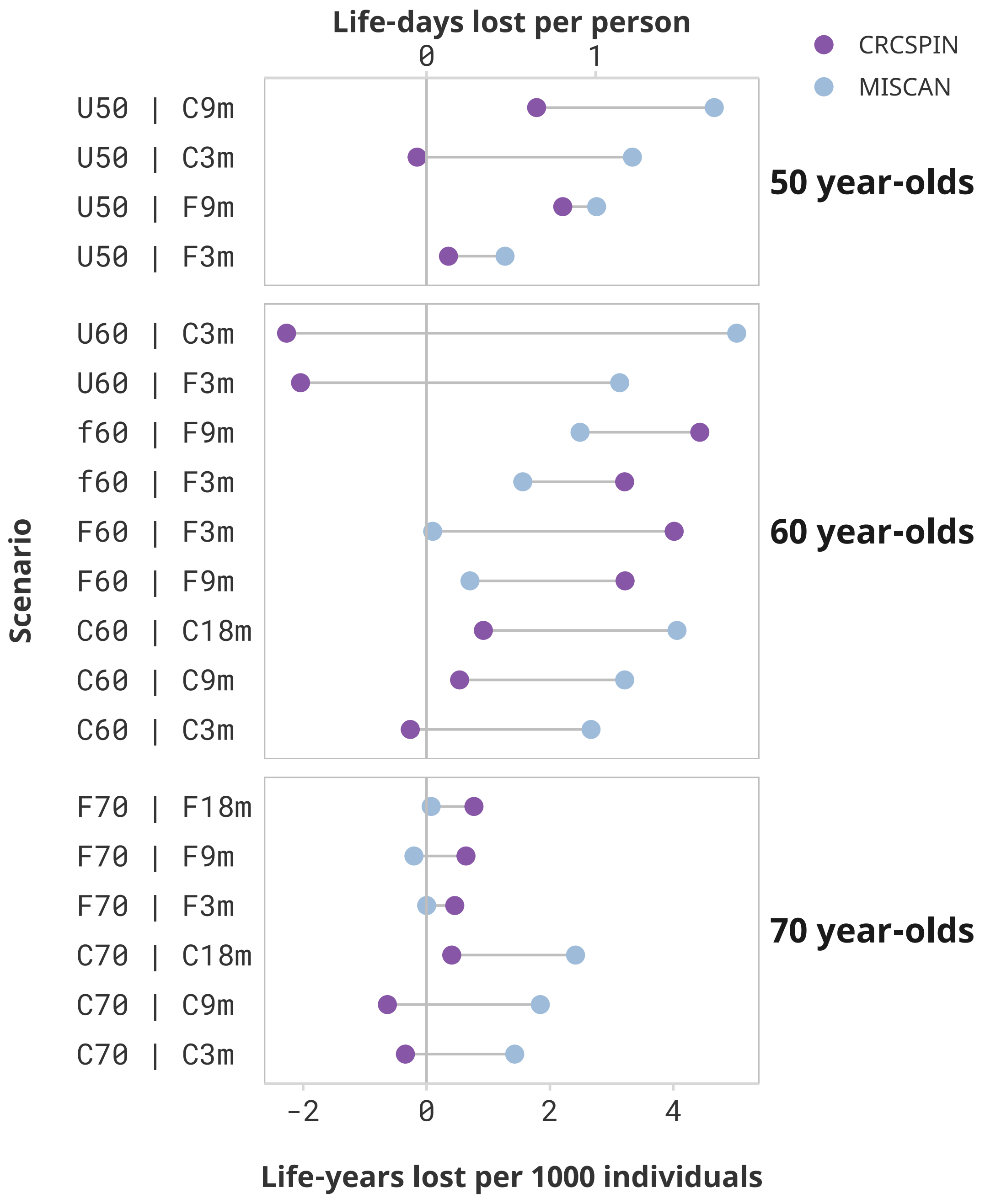
Life-days lost per person
0
1
CRCSPIN
MISCAN
U50 | C9m
U50 | C3m
U50 | F9m
U50 | F3m
50 year-olds
U60 | C3m
U60 | F3m
f60 | F9m
f60 | F3m
F60 | F3m
F60 | F9m
C60 | C18m
C60 | C9m
C60 | C3m
60 year-olds
F70 | F18m
F70 | F9m
F70 | F3m
C70 | C18m
C70 | C9m
C70 | C3m
70 year-olds
Scenario
-2
0
2
4
Life-years lost per 1000 individuals

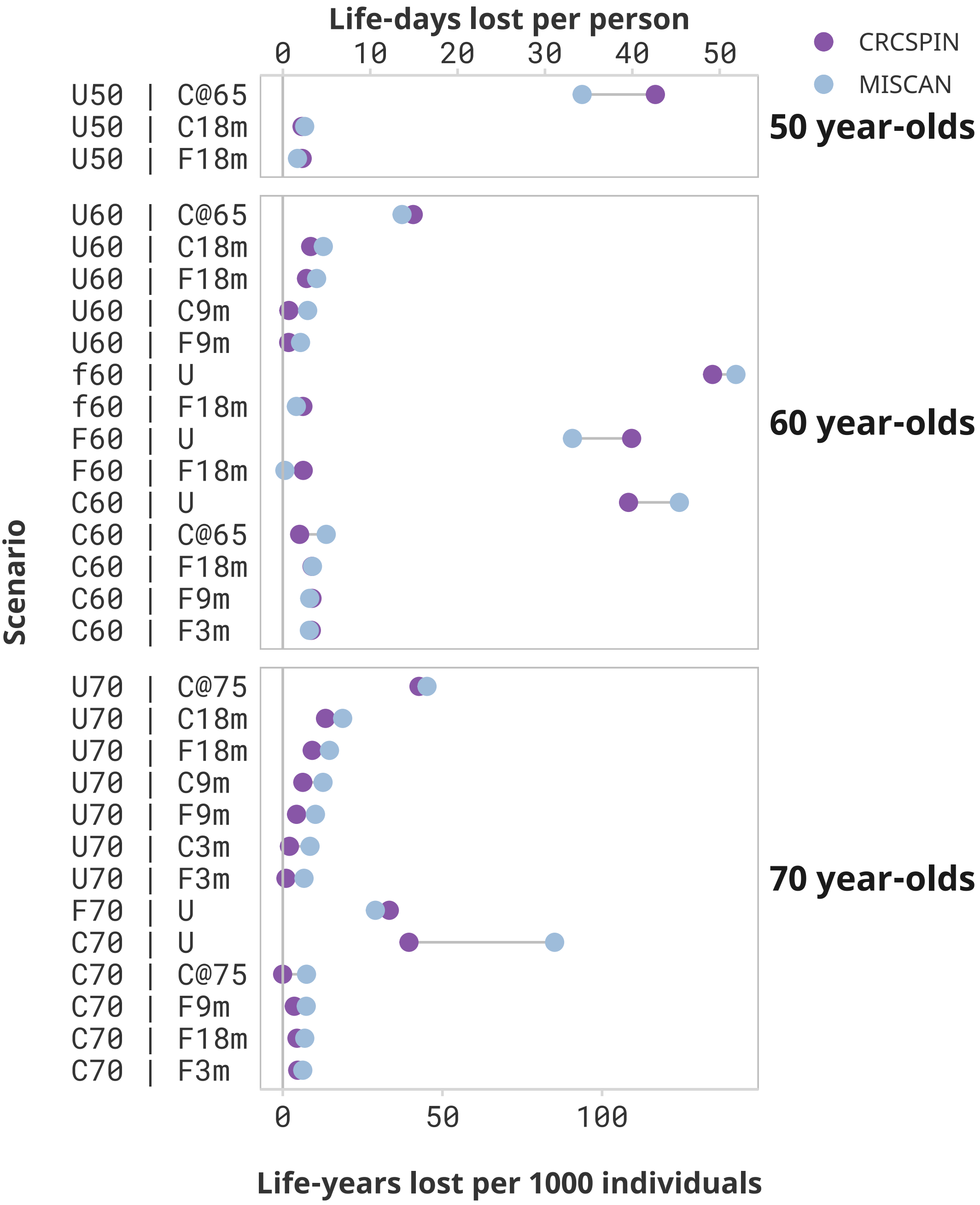

Life-days lost per person
0
10
20
30
40
50
CRCSPIN
MISCAN
U50 | C@65
U50 | C18m
U50 | F18m
50 year-olds
U60 | C@65
U60 | C18m
U60 | F18m
U60 | C9m
U60 | F9m
f60 | U
f60 | F18m
F60 | U
F60 | F18m
C60 | U
C60 | C@65
C60 | F18m
C60 | F9m
C60 | F3m
60 year-olds
U70 | C@75
U70 | C18m
U70 | F18m
U70 | C9m
U70 | F9m
U70 | C3m
U70 | F3m
F70 | U
C70 | U
C70 | C@75
C70 | F9m
C70 | F18m
C70 | F3m
70 year-olds
Scenario
0
50
100
Life-years lost per 1000 individuals

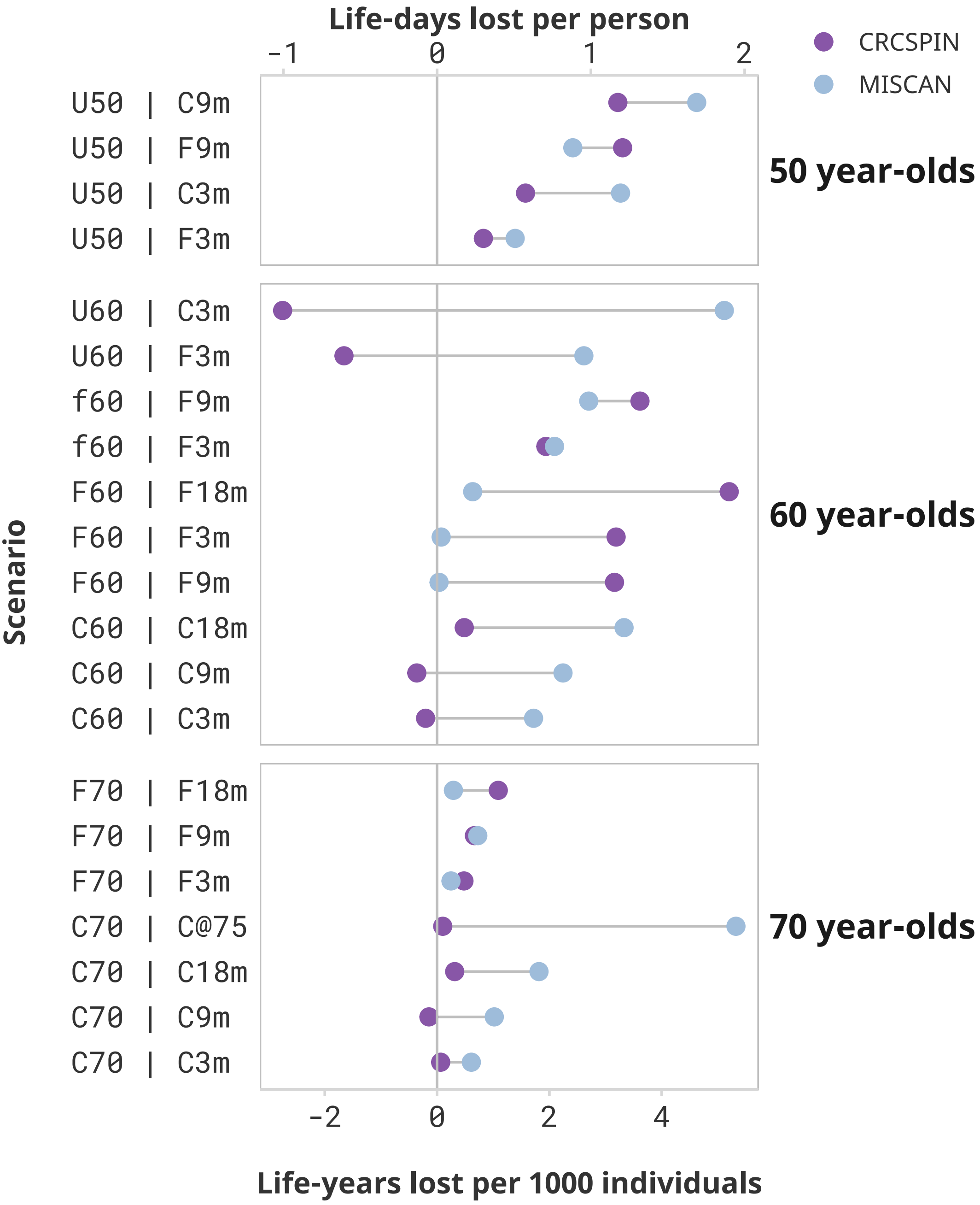

Life-days lost per person
-1
0
1
2
CRCSPIN
MISCAN
U50 | C9m
U50 | F9m
U50 | C3m
U50 | F3m
50 year-olds
U60 | C3m
U60 | F3m
f60 | F9m
f60 | F3m
F60 | F18m
F60 | F3m
F60 | F9m
C60 | C18m
C60 | C9m
C60 | C3m
60 year-olds
F70 | F18m
F70 | F9m
F70 | F3m
C70 | C@75
C70 | C18m
C70 | C9m
C70 | C3m
70 year-olds
Scenario
-2
0
2
4
Life-years lost per 1000 individuals
